# The Mass-Querade: Lung Abscess Mimicking Primary Lung Cancer

**DOI:** 10.7759/cureus.27725

**Published:** 2022-08-06

**Authors:** Olawole Akinboboye, Sheri Walls

**Affiliations:** 1 Internal Medicine, Piedmont Athens Regional, Athens, USA

**Keywords:** imaging, pulmonology, antibiotics, lung cancer, lung abscess

## Abstract

A 68-year-old male with a known history of von Hippel-Lindau disease with brain hemangioblastoma status post radiation therapy and recurrent hemangioblastoma in the spine and multiple spinal surgeries presented initially to the emergency department due to hemoptysis and worsening shortness of breath. A CT chest demonstrated a left lung mass and left pleural effusion, which was initially suspected to be lung malignancy given his symptoms and history. However, it was determined to be a lung abscess following workup and consultations. This case highlights the similarities in the presentation of both pathologies and the critical features in lung abscesses.

## Introduction

A lung abscess is necrosis of lung tissue and formation of cavities containing necrotic debris or fluid caused by microbial infection, most commonly anaerobic bacteria [1]. Lung cancer is the deadliest cancer in men and women worldwide, with about two million cases per year. It has a significantly high fatality rate, so it is essential to identify them as early as possible and start treatment as soon as possible [2]. For identification of such pathologies, A Computed Tomography (CT) scan of the lung is often initially used. The imaging helps classify the lesions and shows metastasis to other organs, if any exists [3]. Though rare, there are specific characteristics of the lung abscess which can mimic and be confused for cancer [1]. We present a case of a patient with several risk factors for lung cancer, CT scan of the lung shows findings suggestive of lung cancer but ultimately found to have a lung abscess.

## Case presentation

A 68-year-old caucasian male with a past medical history of von Hippel-Lindau disease, brain hemangioblastoma status post radiation therapy three years ago, and recurrent hemangioblastoma in the spine and multiple spinal surgeries presented to the emergency department with complaints of hemoptysis, worsening shortness of breath for ten days and unintentional weight loss of 60 lbs over eight months. 

**Figure 1:**
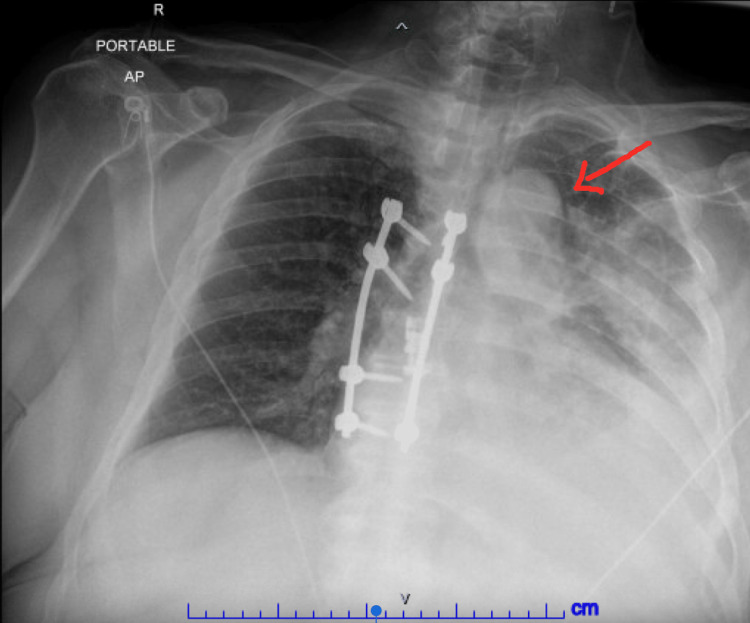
Chest xray revealing left lung mass (Red arrow) with near complete opacification of the left lung

On examination, his vital signs were stable, and his oxygen saturation was 97% on ambient air. Examination of his chest was pertinent for decreased air entry to auscultation and dullness on percussion on the left lower and middle zones. The rest of his systemic examinations were normal. Laboratory investigation showed a white blood count of 11,700 cells/µl and hemoglobin of 10.3g/dl. Chest X-ray revealed near complete opacification of the left lung, likely a combination of atelectasis and effusion, and demonstrated a 4.4 x 5 cm left upper lobe mass as seen in figure 1.

**Figure 2:**
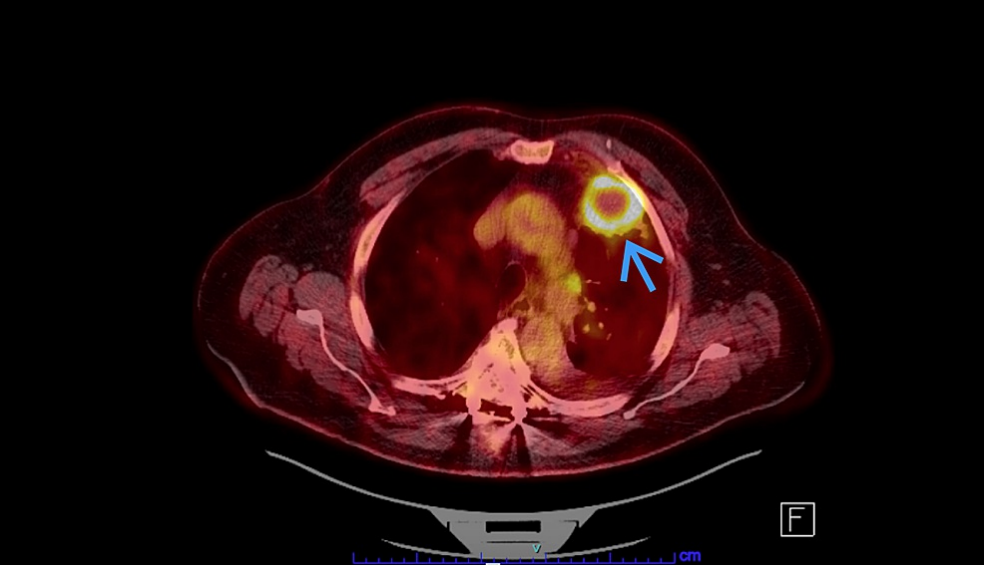
PET CT scan of the chest with intensely hypermetabolic necrotic mass in the anterior left upper lobe (Blue arrow)

Initial differential diagnosis included malignancy versus lung infection. Oncology consultation was done, and the patient underwent a positron emission tomography (PET) scan, which showed an intensely hypermetabolic necrotic mass in the anterior left upper lobe with adjacent satellite nodules along with wedge-shaped moderate metabolic consolidation in the inferior lingula and moderate left pleural effusion as seen in figure 2.

He then underwent a thoracentesis which revealed a transudate with a total protein of 2.9g/dL and lactate dehydrogenase (LDH) of 80U/L, and differential revealed 44% neutrophils, 13% lymphocytes, 5% monocytes, 23% mesothelial, 14% macrophages. Infective etiology was not ruled out. The patient then underwent bronchoscopy and transthoracic needle aspiration by pulmonology. The mass reported on the x-ray was consistent with necrotic debris; cytology revealed neutrophils predominantly with few lymphocytes, macrophages, and bronchial epithelial cells and was negative for malignant cells. Cultures collected showed >10,000 CFU/ml of normal respiratory commensal flora with no Staphylococcus aureus or Pseudomonas. He was then diagnosed with a lung abscess, initially started on ampicillin-sulbactam, and discharged home on amoxicillin-clavulanic acid to complete 28 days of antibiotic therapy. He followed up in the office after four weeks, and his symptoms were significantly improved; however, repeat imaging was not performed, and the reason was not stated in the documentation.

## Discussion

A lung abscess is an enclosed collection of pus leading to necrosis secondary to an infection. It is essential to recognize this due to the mortality rate of up to 38% [2]. Patients at risk for abscess are alcohol abuse, gingival disease, advanced age, vomiting, gastric reflux, and underlying lung conditions such as bronchiectasis. Two types of abscesses can be classified as primary or secondary abscesses. Primary abscesses are the predominant type, carrying 80% of cases caused by patients at increased risk of aspiration. Secondary lung abscesses are caused by underlying conditions such as immunocompromised malignancy and surgery complications [4]. Associated conditions of lung abscess include bronchopulmonary fistula, empyema, and necrotizing pneumoniae. The infection is usually polymicrobial, which could arise from anaerobic and aerobic bacteria. Anaerobic bacteria are usually polymicrobial, which include Fusobacterium, Peptostreptococcus, and Bacteroides. Aerobic bacteria are usually monomicrobial, which include group A Streptococcus, methicillin-susceptible Staphylococcus aureus (MSSA), and Methicillin-resistant Staphylococcus aureus (MRSA), Klebsiella, Pseudomonas, Mycobacterium, Nocardia, and Escherichia coli [2,4,5]. 

Clinical manifestation of a lung abscess varies from fever, cough, purulent sputum, pleuritic chest pain, and weight loss that can occur in an acute or chronic phase. The symptoms for the acute phase last for six weeks compared to more than six weeks for the chronic phase. Physical exams can reveal fever, poor dentition, abnormal breath sounds on lung exam, and clubbing of fingers if underlying lung disease is present. Due to the wide array of symptoms, differentials remain broad, which include empyema, septic emboli, lung cancer, pulmonary embolism, vasculitis, and bronchiectasis. 

Diagnosis is usually made by chest x-ray but could be followed by more specific and practical imaging such as computed tomography (CT) to help distinguish between malignancy and abscesses. Chest x-rays will usually reveal a cavity with an air-fluid level. CT can reveal similar radiographic findings but also allows detection of smaller cavities and malignancies and differentiates abscesses from empyema. In some instances, microbiology and bronchoscopy can be used to tailor management [5]. In the case of our patient, with the aid of Pulmonology, it was noted that the patient had previous imaging results, which most likely revealed a lung abscess due to the nature of the patient's presentation and clinical manifestations. The patient was then treated with antibiotic therapy for six weeks. 

Treatment includes antibiotic therapy, which includes clindamycin 600 mg IV every 8 hours, followed by 50-300 mg orally four times daily. Alternative management includes penicillin plus beta-lactamase inhibitor, metronidazole, carbapenems, and quinolones. The duration of therapy is often treated for six to eight weeks. Surgical management is only necessary if initial imaging suggests a large cavity of greater than 8cm, obstructing neoplasm, antibiotic resistance, and massive hemoptysis [6].

## Conclusions

A lung abscess is an infection caused by several etiologies that leads to a polymicrobial collection of pus. Lung abscesses can be differentiated by imaging such as chest x-ray or computed tomography. The advantage of chest computed tomography is that it allows for better visualization and distinguishes between benign and malignant causes. It is always important to consider the risk factors predisposing patients to both lung cancer and lung abscesses on the first presentation, as some of the symptoms can be deceiving and nonspecific. Management of lung abscesses includes 6-8 weeks of antibiotic treatment. If antibiotic treatment fails, the patient will need to undergo surgical management. 
